# Hydrophobic treatments and their application with internal wall insulation

**DOI:** 10.14324/111.444/ucloe.1978

**Published:** 2024-11-08

**Authors:** Toby Cambray, Valentina Marincioni, Hector Altamirano

**Affiliations:** 1 UCL Institute for Environmental Design and Engineering, London, UK

**Keywords:** hygrothermal, sustainability in architecture and the built environment, solid wall insulation, water repellent, energy and climate, hydrophobic, internal wall insulation

## Abstract

Hydrophobic (or water repellent) treatments have been proposed to mitigate moisture risks associated with internal wall insulation when applied to solid masonry walls. This can reduce risks associated with moisture accumulation within the structure such as mould growth or the deterioration of joist ends and other embedded timber. Where treatments perform well there is a net reduction of moisture content and risk. However, such treatments slow down drying processes, and therefore may result in a net increase in moisture if the treatment is bypassed by, for example, cracks. Some treatments may lead to damage to external masonry surfaces in some situations. Freeze–thaw and salt crystallisation are the two main causes. Hygrothermal simulations may give some indication of risks but techniques to assess the risk of surface damage are either simplistic, impractical outside of the research environment or both. This paper reviews the current field of assessing and predicting the risk of surface damage associated with surface treatments.

## Introduction and context

Demand side energy reduction is an effective way to address the energy trilemma – energy security, fuel poverty and reducing carbon dioxide emissions associated with space heating [1–3]. There are approximately 7.8 M solid wall dwellings in the UK, representing around 30% of the building stock. A significant proportion of these must be insulated as part of efforts to reduce demand, and of those, a significant proportion cannot be insulated externally, meaning that they must be insulated internally [4].

The physical mechanisms which govern heat and moisture in construction materials are interdependent [5,6]. Many of the failures reported with buildings are related to moisture [7] Moisture must be particularly considered when undertaking energy retrofits [7–9].

The moisture safety of internal wall insulation (IWI) presents particular technical challenges [7–9], an important one being the exposure of the insulated wall to wind-driven rain [10]. In contrast to external wall insulation (EWI), IWI does not present an opportunity to reduce rain incident on the masonry. IWI increases the risk of moisture accumulation from the diffusion of vapour from inside which is often, but arguably imprecisely, referred to as interstitial condensation [11]. Also, the addition of IWI causes an average reduction in temperature gradient across the masonry and adds layers to the inside surface which present some resistance to the movement of moisture inwards. IWI thereby reduces the ability of the wall to dry on both sides, which can cause a net increase in moisture content over a typical year [12].

Hydrophobic treatments (HPT), sometimes referred to as colourless water repellents (to distinguish them from paints, renders and film-forming coatings, which are not explicitly considered here), have been proposed as one means to mitigate moisture risks associated with IWI [13–16]. HPT are solutions or emulsions of the compounds which can render a surface hydrophobic. In the context of the built environment, they are applied to masonry to reduce the absorption of rain and therefore mitigate moisture risk. Modern treatments are designed to be applied to existing walls (by spray, brushing, roller, etc.), whereupon they are absorbed into the porous structure (mainly) by capillary suction. The solvent or dispersant evaporates leaving the active ingredients behind which binds to the pore surfaces.

HPT may help reduce moisture risks in masonry walls by reducing the amount of rain absorbed; they have been deployed on walls with cavity wall insulation (CWI) [17], IWI [14,18–20], as well as in the absence of insulation to, for example, arrest/impede weathering in stone monuments and to prevent graffiti. If HPT can mitigate such risks, they may permit the use of insulation in areas (geographical and at building level) where they would otherwise not be possible. They may also permit lower U-values to be achieved with IWI, as risks increase with insulation thickness. HPT may therefore have an important role in reducing energy demand and associated carbon dioxide emissions.

However, deep energy retrofit is prone to unintended consequences, and heritage organisations and practitioners warn of treatments leading to the destruction and loss of historic masonry surfaces [21–23].^1^ There is some evidence to support these concerns; however, the risk depends on the hygrothermal behaviour of the treatment–substrate combination. On the contrary, in some cases treatments have been found to improve freeze–thaw and salt crystallisation resistance [24]. New products appear from time to time, and evidence of failure for one product in a particular situation may not apply for a different product and/or situation. It would therefore be useful to be able to make some prediction or risk assessment of influence of a particular treatment/situation on the likelihood of damage due to frost and salt crystallisation.

This paper provides an overview of the literature on surface water repellent treatments on solid masonry walls and surface damage from frost and salt crystallisation, considering past research, as well as recent developments. Both grey literature and peer-reviewed papers were considered in this literature review, which presents (i) a brief overview of contemporary treatments, (ii) the moisture transfer in treated walls, (iii) mechanisms of surface damage and (iv) approaches to representing treatments in hygrothermal simulation and moisture risk assessment.

## Hydrophobic treatments

This section briefly summarises some key literature regarding hydrophobic treatments, grouped in a series of topics.

### General description

In the context of the built environment, HPT are supplied as a liquid solution or ‘cream’ (emulsion) of active ingredients. Some products are delivered in a concentrated form for dilution immediately prior to use; in this way the concentration can be adjusted according to the substrate and conditions. The product is applied to porous masonry surfaces by spraying, brushing or using a roller and the product is imbibed principally by capillary suction. The solvent or dispersant then evaporates, leaving behind the active ingredients which bind to the internal surfaces of the pores. The molecules are polar, such that one end binds to the substrate and a water repellent ‘tail’ is left exposed. A wide variety of compounds exist that create a hydrophobic effect, including silanes, siloxanes and other silicon-based materials, as well as metal-organic compounds, organic resins and fluorine-containing polymers [25]. Some more complex compounds form extensive matrixes rather than simple linear ‘tails’.

There are examples of damage in masonry associated with HPT [21,26]; silicone-type products, common in the past, tend to block pores and can reduce the ability of materials to dry out or ‘breathe’,^2^ as some say [8]. This has been put forward by some practitioners as the reason for discouraging the use of these treatments [22]. However, these conclusions may be based on treatments used in the past, which remain on the external surface rather than penetrating into the pore structure.

The chemistry of treatments has advanced significantly in the century since some of the basic molecules were first synthesised [25]. Due to their ease of application and the absence of organic solvents (which have environmental implications), water-based emulsion type products are increasingly popular [27–30], but several other types of compound can create a hydrophobic effect [25,31]. Improved chemistry has led to at least some products which block the pores to a lesser extent and therefore reduce the vapour diffusion resistance by only a small proportion [31,32].

Finally, it is worth noting that there are often similarities (chemically and functionally) between products intended to serve as water repellents, and those classed as consolidants, which are intended to strengthen masonry – usually natural stone – by replacing lost or weakened minerals. Additionally, they are sometimes used together in a bid to a) halt deterioration and b) to prevent further degradation [23,25,33]. The discussion here will focus on products intended for use as HPT rather than consolidants.

### Water repellent treatments in practice

Charola [25] gives a brief history of surface treatments through the 20th century and discusses the chemistry of silicon-based treatments, as well as the importance of the formulation, application and substrate; and the durability, re-treatment and negative effects of treatments. Weeks et al. [34] give a practical and detailed overview of the products available to the UK market. Soulios et al. [31] investigate the hygrothermal properties of a range of European products when applied to various porous materials, giving data on the change in absorption and vapour resistance. Van Hees [23,35] undertook an extensive study in Belgium, Italy and the Netherlands covering 60 brick buildings which had been treated in the past. While the records and information about the treatments was usually missing, the treatments appear to be long-lived [23,35]. van Hees’ study covers several important topics – field and lab measurement techniques are developed for the assessment of hygrothermal performance, consumption rate and impregnation. All these factors are found to depend on the combination of treatment product and substrate. Artificial weathering including salt crystallisation is discussed and methods proposed. Lubelli presents a state-of-the-art method for artificial weathering and salt durability based on a two-step process of salt accumulation followed by damage propagation, which could be applied to materials with HPT [36].

### Simulation work

Much research has been undertaken via simulation. Comparing simulations with a field trial shows that HPT can result in a lower dynamic equilibrium moisture content, despite a reduction in drying rates. The relative timing of interventions (IWI and HPT) and moisture content is therefore important [15]. More recent work has shown the timing of treatment relative to the installation of IWI has an impact on the thermal performance but long-term moisture safety is improved irrespective of timing [37]. It has also been shown by simulation that HPT can make the difference between an acceptable and unacceptable level of risk associated with IWI [38]. Where HPT (without IWI) results in a drier masonry wall, the thermal performance is improved due to masonry materials having a thermal conductivity that increases with moisture content [39]. Introducing any material to the pore structure will reduce the porosity and therefore increase the vapour resistance, but in the case of a German sandstone, there is no increase in moisture content for modest increases in vapour resistivity [40]. It has been proposed that a partially effective HPT may provide a useful balance of positive and negative impacts [16]. Several simulation studies explore the hygrothermal performance of one, or a small number of masonry materials, IWI and treatment combinations [19,38,41].

### Laboratory work

HPT have also often been studied in the laboratory. Several techniques such as X-ray diffraction, Fourier transform infrared and gas chromatography can be used to infer the composition of products [30]. Depth of HPT penetration is an important parameter, and it can be measured destructively [42] and non-destructively with micro-computed tomography techniques [43]. A variety of products, on a variety of substrates, has been tested in the UK context [32] and in the mainland Europe context [31,44]. In such work it is typical to represent performance via change in absorption, measured by partial immersion [31,44] or Karsten tube [30]. The influence of treatments on vapour permeability is variable, and likely to depend on the particular treatment/product combination. It is typical for the smallest pores to become blocked [44,45].

Lab techniques to apply liquid products to small specimens often differ significantly from in-situ methods; in the lab, partial immersion is typically used for its convenience, but this can yield different depths of penetration compared to spraying as is typical in real situations. Furthermore, the influence of mortar can be significant and the depth of penetration can vary significantly, due to the different absorption characteristics [42]. Silane-only treatments have been compared with silane siloxane blends; the former yield less strongly hydrophobic treated zones when applied at the manufacturer’s recommended rates. This results in a less dramatic impact on drying behaviour [46]. The role of cracks in bypassing the treated zone is important; cracks of more than 0.1 mm may be compromised during moments of high wind pressure with flow continuing after the gust has passed [47]. This effect can also result from capillary condensation [45].

Case studies of using HPT on real buildings or full-scale assemblies include [14,17,18,20,21,26,35,46].

### Longevity

The longevity of treatments is also sometimes questioned. As with many aspects of the performance, the answer depends on factors such as the treatment itself, the substrate and conditions of application. A multinational research project including in-situ case studies found that treatments can be expected to provide a hydrophobic effect for significant periods, [23,30]. One laboratory study using accelerated weathering with simulated rain and ultraviolet (UV) exposure showed that while the beading effect on the exposed surface may deteriorate (probably due to UV degradation of the exposed product [48]), the treatment below the surface is not impaired and the absorption coefficient in fact reduces [27]. On the contrary, other researchers find a gradual decline in performance with limestones under accelerated weathering [30]. It could be concluded that testing of proposed product/substrate combinations should be undertaken prior to treatment, including accelerated weathering, wherever practical.

### Moisture transport in treated materials

#### Moisture transport and storage in hydrophilic and hydrophobic porous materials

##### Wetting

Moisture can enter a porous masonry wall by many different routes, which can be categorised into: as-designed, theoretical (ADT) and as-built, in-service (ABIS) conditions.

A key difficulty in hygrothermal simulation is to make meaningful representations of such particular conditions, especially where they may or may not occur in the future [7,49].

The main objective of hydrophobic treatments in this context is to prevent the capillary absorption of rain on walls. As the majority of the moisture in a solid wall is due to wind-driven rain [10], this might seem an effective strategy. Furthermore, simulation shows that as long as the vapour permeability is reduced (by the treatment) by less than 25%, moisture diffusion from the inside will not cause a significant increase in moisture content, even if the internal relative humidity (RH) is increased [40]. This suggests that it is sources of liquid water ingress that should be of primary concern.

However, moisture can enter wall constructions by routes other than rain absorption at the surface and diffusion from inside. Indeed, where water is the carrying-agent of treatments the application of treatment itself has the potential to introduce significant levels of moisture [20].

While it is trivial to observe that a leaky drain is likely to cause problems and is therefore unnecessary to simulate numerically, there are smaller compromises such as imperfections in pointing which may or may not immediately lead to failure. Allowing for some degree of moisture ingress therefore allows for the testing of the resilience of a wall or other element [49]. This approach is aligned with the four C’s: context, coherence, capacity and caution [7].

In particular, cracks can play an important role in the ingress of water through surfaces that have been treated. In the case of a crack which occurred before treatment, the internal surfaces can be expected to be hydrophobic, and the behaviour of cracks can be conceptualised in a similar way as a hypothetical capillary tube. The negative hydrostatic head that occurs where there is a contact angle less than 90° can in principle be overcome with a sufficient wind-induced pressure [47]. Significantly, with respect to simulation, gusts and the momentary increases in pressure play a critical role in infiltration through cracks; typically, climate data used in hygrothermal simulation is based on hourly averages and does not reflect momentary peaks in windspeed. If the wind pressure is sufficient to overcome the capillary pressure, even momentarily, water can ‘bridge’ through the crack, essentially forming a meniscus either side of the crack that extends to a point deep enough into the crack to make contact with a hydrophilic surface [45,47].

In the classic capillary tube experiment [50], the opposing forces are the (negative) capillary pressure and the weight of the column of fluid; hence an equilibrium height is achieved. In the case of a (hydrophobic) crack, the main opposing forces are the (negative) capillary pressure, and the static wind pressure. The depth does not influence the resistance to ingress with respect to wind speed because flow through the crack will initiate as soon as the hydrostatic pressure exceeds the capillary pressure [47]. This means that an increased depth of penetration does not necessarily protect against rain ingress through cracks, although the nature of the crack in terms of shape and depth is also crucial. Surface cracks that do not extend beyond the treated zone will not in principle result in rain ingress.

Where a ‘bridge’ through the hydrophobic region is set up, it will persist while there is a supply of liquid water from the surface, even after the gusting pressure has passed. During a storm, a crack might be repeatedly bridged and emptied. Such bridges can also be formed as a result of capillary condensation [45].

Manufacturers typically recommend that cracks greater than 0.3 mm are remedied before the application of treatments [47,51], but a maximum crack size of 0.1 mm might be more appropriate, considering realistic wind pressures and contact angles [47]. The static wind pressure which a hydrophobised crack will resist depends on the width of the crack and the effective contact angle; narrower cracks with a higher contact angle (more hydrophobic) resist higher pressures. This behaviour in cracks in consistent with observations that in some cases rain ingress though masonry leaves (as distinct from absorption into the materials) can be increased by hydrophobic treatments [52].

Where moisture is present in the ground in sufficient concentration near to foundations (irrespective of its root source) it will be drawn up into walls without effective damp-proof courses. A simple ‘sharp front’ model shows that the height of the drying front is strongly dependent on the relationship between the rate of wetting and the rate of drying. The latter depends on the height, which simply increases the area over which drying can take place [53]. Hydrophobic treatments tend to reduce the rate of drying (as discussed below), and hence will tend to increase the height of rising damp and the total average moisture content of a wall. This is one mechanism by which inappropriate treatments could lead to an increase in moisture content of walls.

Additionally, ground water is one of several sources for soluble salts, which are important agents of deterioration of the surfaces of masonry [54–56]. The interaction between hydrophobic treatments and salts is discussed in section Cryptoflorescence below.

##### Drying

Drying of (hydrophilic) porous media is a topic that is relevant to various fields including manufacturing and agriculture and has been thoroughly studied.

When a porous material is at or near to saturation and in the process of drying, a film of water can be maintained on the surface by internal liquid transport processes drawing on water stored in the pore structure below the surface. This is widely referred to as Stage 1 drying [57,58].

At some point during the drying process, the rate of internal liquid transport falls below that of the surface evaporation. This point marks the transition from Stage 1 to Stage 2. Stage 2 is limited by the internal transport processes, and the importance of vapour diffusion increases. Whereas in Stage 1 the rate of drying is constant (for constant boundary conditions) in Stage 2 the rate of drying is inversely proportional to the square root of time [57–59].

### Hydrophobicity and reduction of wetting

#### Fundamental theory

Hydrophobic surfaces have a water contact angle greater than 90°, meaning that water will ‘bead up’ and roll off surfaces. HPT work by modifying the surface energy of the internal surfaces of the porous substrate to which they are applied. Typical, untreated masonry materials are categorised as hydrophilic [12,28].

The high contact angle means that, in theory, a material that has been treated such that its surfaces might not be expected to exhibit any capillary suction. Considering a porous material which is entirely hydrophobic (i.e., all the surfaces have a contact angle with water more than 90°), the meniscus will be inverted, and water can only enter the pores under a positive pressure. Furthermore, the pressure required to overcome the ‘negative suction’ is inversely proportional to the pore diameter. A simple analysis would therefore predict that there is no liquid transport in a material which has received a treatment with water repellent, and that if the cross-sectional area of the pores is not significantly reduced the passage of vapour will remain unimpeded [48].

#### Experimental results on hydrophobicity of treated walls and masonry materials

Contrary to this basic theory, experiments to measure liquid conductivity generally find after treatment a much reduced, but still measurable degree of absorption after treatment [28,31,46] (Rirsch, 2010).^3^ There are several possible reasons for this:

1. Carmeliet et al. [45] observe that in smaller pores, the size of the active molecules is such that some treatments are more likely to block the pores, or not access them at all. This is reflected in experimental results [44]. These effects may contribute to changes in observed vapour permeability, as well as the observed residual liquid suction.
2. A temporary reduction of waterproofing was also found for water-based emulsion treatments. These products use surfactants which are necessary to create the emulsions. To do so they are effectively hydrophilic agents; in other words, they counteract the hydrophobic effect of the compounds they help maintain in suspension. The surfactants do not bind to the substrate and dissipate over a relatively short period of time with the action of rain, in the order of months perhaps. They tend to accumulate near the surface from which the treatment dries out. While they persist, there is a reduction in hydrophobic effect.^4^
3. The observed changes in vapour permeability and liquid absorption could be due to a number of factors, several of which could interact. The test methods codified in international standards are necessarily practical and applied in nature and cannot entirely differentiate between liquid and vapour processes. Test methods generally reflect the conceptual division between liquid and vapour processes in porous materials. While this distinction is useful in many ways, it is a simplification and there are mechanisms that operate in a ‘grey area’ between and across the liquid and vapour domains, in particular drying processes [59]. Such mechanisms become more important when considering certain aspects of the behaviours related to HPT.
4. Considering typical partial immersion tests for liquid absorption, these must necessarily take place adjacent to liquid, from which a specimen will adsorb water due to its hygroscopicity [60]. Typical gravimetric methods for observing the uptake of water cannot distinguish between water imbibed by capillary suction and that which is adsorbed hygroscopically [61]. A significant degree of hygroscopicity remains after treatment [45] and depending on the objective of the test and the application methods of the lab, there may be an untreated zone in the specimen. Some of the mass increase observed in the absorption experiment may therefore be due to hygroscopic sorption and capillary condensation, although this is likely to make only small differences especially over the short time periods over which the majority of absorption occurs, compared to the time necessary to accurately measure the hygroscopic sorption. To minimise this effect, test methods call for specimens to be in equilibrium with the lab environment [60], but the air above the test bath may be at elevated RH. Furthermore, the air in the pores may be subject to moisture evaporating (and indeed, diffusing) ahead of the wetting front.
5. Similarly, liquid transport and storage cannot be entirely excluded from vapour transport experiments and tests. A typical approach is to fix a specimen over the top of a cup containing known saturated salt solution, such that a vapour pressure gradient is established and controlled [62]. At all non-zero water vapour pressure, a porous specimen will exhibit hygroscopic sorption and at higher RH, capillary condensation will occur irrespective of treatment, which may lead to some liquid redistribution [45]. It should, however, be noted that in these measurements the liquid transport does not compromise the validity of the measurement (in contrast to reason 4). It only highlights the artificial separation of liquid and vapour transfer. Additionally, the typical decrease in water vapour resistance of materials at higher moisture contents may be less evident for materials with HPT.

These observations go some way to explaining the small amount of absorption that is routinely observed after the application of HPT.

### The influence of hydrophobic treatments on the moisture balance

#### Wetting

The purpose of hydrophobic treatments is to reduce the capillary suction of a porous material and thereby significantly reduce the amount of wetting of the materials from rain incident on the façade. Most contemporary treatments are very effective in this regard, as measured by the reduction in absorption by partial immersion experiments [31,44,46], notwithstanding moisture that may bypass the treated zone by one of the mechanisms discussed above. Not all treatments are compatible with all substrates, and where there is incompatibility, performance is compromised, either over time or from the outset.

#### Drying

Where HPT have been applied, the liquid transport in the treated zone is reduced, generally to negligeable levels. This means that Stage 1 drying is effectively precluded [40,44,46,48]. Stage 1 drying is faster than Stage 2, and so preventing it reduces the rate at which a material can dry, from what is potentially its most vulnerable state, that is, at or near saturation.

These observations agree to some extent with two seemingly contradictory statements put forward on one hand by advocates of treatments, and opponents on the other:

- Treatments ‘trap’ moisture in masonry walls
- Treatments are ‘breathable’

These two statements are somewhat unscientific, but typical in practise and sales literature. Firstly, while drying is possible with many types of treatments (i.e., those with a modest or negligeable impact on vapour permeability), there is a significant reduction in drying due to the prevention of Stage 1, which is inevitable due to the purpose of the treatment. Secondly, if we take ‘breathable’ to mean simply vapour-open, many modern treatments have only a small impact [31,32,46]. An important variable with respect to the hygrothermal performance of treated walls is the depth of penetration of the treatment into the substrate, and thereby the thickness of the region which is hydrophobic.

Several researchers have observed regions with variable efficacy occur with some products and substrates [31,42,46]. Typically, a shallow region near the surface (up to a few mm) is highly hydrophobic, with a deeper less strongly hydrophobic region (up to 50 mm). This is likely be associated with the application of multiple coats, which is not relevant where the product is formulated to be applied in one coat. This variable could have a significant impact on drying behaviour as this is directly linked with the thickness of treated material through which vapour must diffuse in Stage 2. The meaning of ‘strongly hydrophobic’ in this context is usually framed in terms of contact angle or reduction of liquid absorption, but this may not be an adequate definition for the same reasons as discussed above in relation to residual liquid transport [45,63].

A deeper hydrophobic region may give more protection to some types of ingress, but this is not always the case as discussed above with respect to cracks. Conversely, a deeper hydrophobic region will present a higher vapour resistance and retard drying. Furthermore, some mechanisms of failure act at the boundary between hydrophobic and hydrophilic regions; where this occurs, a deeper hydrophobic region will result in loss of more material.

### Moisture risks and mechanisms of failure

#### Overview

Water is an important agent of decay and some (but not all) of the problems associated with it can be expressed as a matter of ‘too much water in the wrong place’. This applies to phenomena such as mould growth and the decay of timber [64,65]. In this sense, moisture risk can be conceptualised as a disturbance to the balance between wetting and drying, leading to inappropriate levels of moisture [7].

In the case of IWI applied to a solid masonry wall, the risks include accumulation of moisture at the critical interface (i.e., between the insulation and the masonry), possibly leading to the growth of mould [66]. Where the insulation is biodegradable (e.g., wood fibre) there may be a risk of rot and destruction of the material. Embedded timbers such as built-in joist ends may similarly be vulnerable [20,65,67]. These risks are reasonably well understood and routinely assessed with the aid of hygrothermal simulation. Temperature and humidity can be estimated via numerical simulation and used to inform an assessment of these risks [68–70].

If an HPT is used and is effective, it might be expected that the wall is generally drier than without it, and therefore at a lower risk. If there is bypass of the treatment, the risk of mould and rot will depend on the extent and position of the bypass, which is practically impossible to predict, but can be allowed for in a basic manner [71].

A second group of risks is those which involve damage to the external surfaces. The two most important are freeze–thaw or frost damage, and damage due to the expansion of salt crystals; these are at least the most common in the literature. In frost damage, the expansion of ice creates stresses and strains that overcome the masonry [72–74]. Salt crystallisation and hydration occurs when salts similarly expand and push materials apart from within [54,75–77].

It is also commonly observed that inappropriate re-pointing of historic lime-rich mortars with cement-based materials can lead to damage to the masonry units. It is generally agreed that historic pointing should be regarded as semi-sacrificial, and if relatively weak pointing is replaced with higher strength cement mortar which also has lower liquid conductivity, drying occurs preferentially via the masonry units which can suffer damage as a result. This may be due to freeze–thaw or salt expansion [78,79].

A further mechanism, which is less frequently mentioned in the literature, is differential hygric expansion. It is known that certain types of stone and brick (in particular clay-rich materials) expand with increasing moisture content. If there are differences in moisture content within the same monolithic piece of material, internal stresses and strains will be set up [25].

#### Freeze–thaw

Freeze–thaw damage is an intuitive mechanism of failure. While this mechanism is relatively simple at a superficial level, the details become very complex, and it is impractical to predict this type of damage with any degree of certainty with the current state of the art.

An important variable is the degree of saturation. If there is a small amount of water in the pore structure, it will simply expand into the space occupied by air, displacing the air but without exerting forces on the insides of the pores. As the moisture content increases, there is less and less space for water to expand and so it is more likely that forces will be generated. This can be expressed as a degree of saturation, each material having a different critical saturation point, below which damage does not occur [80]. This effect is further complicated by the pore size distribution; at equilibrium the moisture will occupy smaller pores preferentially.

The complexity of interaction with pore size is compounded by the fact that the freezing point of water is influenced by the pore structure. The freezing point is lower within smaller pores, although this effect is most significant below approximately 0.1 μm, which is less important for liquid transport [74,81]. The presence of salts also reduces the freezing point of water. There is a strong inverse correlation between total porosity and frost resistance, that is, more porous materials are less frost resistant. However, the proportion of pores smaller than 3 μm is strongly positively correlated with resistance, that is, a material with pores predominantly below 3 μm is more frost resistant, even if the total porosity is high [82].

With respect to the influence of hydrophobic treatments, the net impact on risk hinges upon the balance between wetting and drying, potentially at highly local level, for example, near a particular crack. The depth of penetration is likely to be significant because the thermal resistance and diffusivity of the mostly dry hydrophobised zone will reduce the amplitude of temperature variations at the depth where moisture content can reach levels necessary for damage to initiate. However, the nature of the reduction in Stage 1 drying means that frost damage risk may be disproportionately increased because the freeze–driven rain thaw risk depends on simultaneous ‘zero-crossing’ and elevated moisture content [72,74].

#### Cryptoflorescence

Cryptofloresence is the growth and expansion of salt crystals below the surface of porous materials. This growth can in some cases generate stresses and strains sufficient to cause damage.

The role of salts in the deterioration of masonry is a large and important field of research which has been active for decades [76]. Research is ongoing and like frost damage there is a gap in the knowledge with respect to the ability to risk-assess future damage from salts. Two principal mechanisms for decay have been hypothesised: crystallisation and hydration [54]. Both involve the expansion of a foreign material (salts), which can induce internal stresses that in some circumstances are sufficient to overcome the tensile strength of some materials.

Efflorescence is a common phenomenon whereby salts are transported, dissolved in water, to the surface of a material as it dries out. At the evaporation surface, the concentration of salt increases because the water evaporates leaving the salt ions behind, which form a crystalline structure. On the surface this is largely an aesthetic problem. In some circumstances, however, crystallisation occurs below the surface. In this case, the growth of crystals can exert extremely high forces on the internal surfaces of pores [55]. This also means that the position of the crystallisation depends on the stage of drying; Stage 1 leads to efflorescence and Stage 2 to cryptoflorescence.

There is some evidence to indicate that hydrophobic treatments promote the more potentially damaging cryptoflorescence over efflorescence [83,84]. This is thought to be because HPT effectively force porous materials to dry entirely in Stage 2 mode, meaning that evaporation occurs below the surface, especially at the hydrophobic–hydrophilic boundary, and crystallisation occurs at the position of evaporation.

A significant research project was undertaken to ascertain the compatibility of surface treatments (not only HPT) with salt-laden masonry. Thresholds have been proposed that vary with the substrate and type of salts present [85].

### Simulation and risk assessment

#### Overview

Hygrothermal simulations using methods such as those compliant with BS EN 15026 (2007) are important tools for assessing moisture risks and may have a role in the process of evaluating whether a treatment should be applied. To undertake hygrothermal simulations of assemblies which include HPT, it is necessary to represent the treatments with sufficient accuracy in the simulation [37]. However, as discussed above, there are significant limitations in the capacity of hygrothermal simulation to predict important risk mechanisms, in particular those associated with the surface damage which may be exacerbated by HPT in certain circumstances.

There are two main categories of methods for representing hydrophobic treatments in simulation: by adjusting boundary conditions, and by adapting material properties. In the latter case, either of a whole leaf of masonry or a thin layer corresponding to treatment penetration appear in the literature. The means for adapting the material properties depends on the working principle of the material model.

The main objective of hydrophobic treatments is to prevent the ingress of rain. With zero air movement, raindrops would fall vertically, and therefore not interact with a vertical wall. Rain is usually affected by wind, which blows it onto walls. This is complex to predict due to the turbulent flow of wind, which depends on a number of site-specific factors.

The ‘catch ratio’ of wind-driven rain is very complex, depending on building geometry and that of the surrounding environment, the subsequent airflows and the rain-drop size distribution. Another significant factor is the capacity of the masonry surface to absorb rain impinging on it and the nature of the run-off.

Zhao and Meissener [86] assume a rain exposure coefficient of 0.3 in an attempt to capture all of the above effects and that of an HPT. This value is an arbitrary reduction that could be due to a number of factors including, for example, overhanging eaves as well as HPT. A significant limitation is that it may overestimate the drying rate where HPT have been applied, because the transport of liquid to the surface is not reduced in the simulation.

An alternative to the simple modification of the boundary condition is the modification of the hygrothermal properties of the material in the simulation. This can apply to the whole of a material layer, for example, an entire 100 mm brick leaf [28]. or modifying a thinner section on the surface corresponding to the depth of penetration of a treatment [37,40,41].

Arregi and Little [41] assess the risk of moisture related problems with an IWI design for a particular house. To represent the hydrophobic treatment, they create a separate layer/sliver of material at the outer surface, with a thickness of 10 mm, corresponding to the absorption coefficient, vapour transmission and depth of penetration observed by Rirsch (2011).^3^ Their analysis shows that for the proposed insulation approach, a hydrophobic treatment can reduce moisture risks associated with IWI, although the simulations do not allow for any liquid water bypassing the hydrophobic coating.

#### Limitations of simulation

Hygrothermal simulation has been shown to be a useful tool in assessing moisture risks in many situations. These techniques should be regarded as indicators rather than absolute predictions because the behaviour in reality depends on factors which are not represented in simulation, or are too site-specific, complex or stochastic to be meaningfully represented. As such a balance must always be struck between on one hand practicality and utility, and on the other accuracy and rigour [11,49].

#### Wetting

All three methods for representing HPT in simulation reduce the wetting from wind-driven rain. In reality there is almost no absorption on the surface with an effective treatment [31,44], although the problem of improper application (too much, too little or patchy application for example) is little studied. None the less, the efficacy should be reflected by reducing the absorption coefficients of a sliver corresponding to the depth of penetration, as long as accurate characteristics are known for the treated and untreated materials. The boundary condition method is less certain because the water repellent surface may not be well-represented by a simple reduction in wind-driven rain.

It is good practise to include additional moisture sources or loads in simulation, and situations with HPT are no exception [7]. A more challenging question is what level of ingress is appropriate? There is no quantitative answer to this currently, although a number of factors should be considered.

Transport of water through hydrophobic cracks could be significant [47] and significantly depends on peak gusting wind pressure; typically, hygrothermal simulations use hourly average wind data which obfuscates the peak speeds. The value of 1% of wind-driven rain ingress is widely used in commercial and academic simulation work [71] intended for use with rainscreen cladding facades, and this approach may or may not be valid in other situations. Researchers and practitioners should consider peak speeds (which may depend on highly localised funnelling effects for example) and adapting simulations accordingly, for example, specifying rain ingress as some function of peak wind speed. A review of research on this topic provides researchers and practitioners with a useful guide to more sophisticated approaches [52].

#### Drying

The boundary condition approach tends to underestimate the moisture accumulation and therefore moisture risk, because it still allows liquid to be transported to the surface during Stage 1 drying. Conversely, the method of adapting the material properties to represent the reduced absorption will reflect the prevention of liquid transport to the surface and the more rapid Stage 1 drying. However, to create an accurate representation, it is necessary to know the specific material properties with and without the specific treatment, and the typical depth of penetration [31]. To reflect the drying behaviour accurately, the depth of penetration is very important, and this will depend on the physio-chemical interaction between treatment and substrate at the point of application.

## Conclusions

HPT may have a role in reducing energy demand and associated costs, insecurities and environmental impacts. They are already deployed in the UK and elsewhere to mitigate moisture risks of IWI by reducing the absorption of wind-driven rain.

There is a possibility of unintended consequences, in particular irreparable damage to historic masonry surfaces. This may result from an accumulation of moisture if the treatment is bypassed via cracks in the surface, ground water or other leaks. The possibility of damage associated with salt crystallisation has also been identified. With existing knowledge and techniques, it is difficult to accurately assess these risks.

Several approaches to representing HPT in simulation have been investigated, and the method of representing the hydrophobic zone as a sliver of separate material corresponding to the depth of penetration is most representative.

If the wetting behaviours can be meaningfully represented, established techniques for the assessment of risk of mould and timber decay should be as reliable as in cases without hydrophobic treatments. However, there exists a group of hygrothermal risks associated with damage to masonry surfaces for which robust risk assessment methods are still being developed, specifically freeze–thaw and salt crystallisation damage.

Hydrophobic treatments often interact differently with mortar than with the brick or stone it bonds. Given that historic masonry often relies on the differential hygrothermal behaviour of mortar and masonry units to manage the risk of surface damage, further work exploring the three-way interaction of mortar, masonry unit and treatment would be valuable. Further evidence from real cases where surface damage has occurred would deepen the understanding of what leads to such problems and would inform research questions. Developments in the areas of predicting or risk-assessing frost and salt damage in the absence of treatments could be applicable to situations where they are deployed. A key challenge is finding ways to make generalisations about situations where there are many factors with case-specific parameters. It is not possible to say a particular product is safe or not, as the risk depends on the substrate and conditions.

## Notes

1 Henn M, Kent D. Colourless water repellants (personal communication). 2021.

2 The term ‘breathable’ is arguably imprecise and open to interpretation; therefore, it is not used further in this paper.

3 https://static.safeguardeurope.com/downloads/reports/stormdry/stormdry-masonry-protection-cream-water-uptake-report.pdf.

4 Rirsch E. Surfactants (personal communication). London: University College London; 2022.
